# The American and Colonial Journals: Pathology, Practical Medicine, and Therapeutics

**Published:** 1839-10

**Authors:** 


### PATHOLOGY, PRACTICAL MEDICINE, AND THERAPEUTICS.

### *Therapeutic Effects of Tobacco applied externally.*

By Alexander Somervail, m.d. of Virginia.

I have suffered from diseased nerves in various ways. Some, such as I have never heard of, and which has introduced an application that I think may be useful in many distressing circumstances. The points of the two middle toes on each foot are affected with a burning sensation, increasing every afternoon, rendering washing painful, sometimes much so with a sense of swelling and weight, very unpleasant; the healthy feeling gone; this threatens my fingers too —when looked at, the point is a little red and painful to touch: it came into my mind to apply a snuff-plaster; this produced immediate relief; but I cannot leave it off for more than a week before the burning returns. Now one toe only resists this application, but is much relieved by it. I have often used this where I used to blister; lately I applied a twist of tobacco softened by boiling, but so as to retain the water to the side, in a case of pain in the side, with cough, fever, &c. in an aged woman, with speedy relief, and by the help of other remedies, recovery followed where I had no right to expect it. The tobacco applied thus is more powerful than the plaster. I have used this since the fever of 1815, occasionally—

no nausea, &c. is produced, unless the cuticle is abraded; in that case we cannot use the tobacco either way. I had a lady last June with severe pain in the face over the antrum; the plaster removed it, and was worn a couple of weeks. Perhaps tic-douloureux may be relieved by it. A negro man, in hewing timber, struck the corner of his axe into the inside of his knee, which lamed him for two months. When I saw him the cut was healed, the knee enlarged, the leg bent, very painful, and could not be extended. The cut appeared to have touched the tendon of the flexor. I applied the plaster; the pain soon mitigated; he got better daily, and soon admitted the use of a splint to extend the contracted muscles and is well.

The snuff-plaster was first prepared by using snuff in place of cantharides, in the preparation of blistering plaster; but as that is not adhesive enough, I have left out the wax in that plaster, and in its place put in empl. lytharg.

*American Journal of Med. Sc. Feb.* 1839.

---

*Account of the Asylum for the Relief of persons deprived of the use of their Reason, near Frankford, Pennsylvania, with the Statistics of the Institution from its foundation* (*May,* 1817) *to the end of* 1838. By CHARLES EVANS, M.D., Attending Physician to the Asylum.

[THIS is a valuable document, and happily illustrates the effect of the improved mode, now so generally adopted, of treating the insane. This Asylum belongs to the Society of Friends,* and was expressly instituted in imitation of the Retreat at York: it is the highest praise we can give it to say that it is worthy of its admirable model. Our limits will permit us to extract the principal statistical details only, and a few extracts from the description of the building, and the general mode of treating the patients.]

The centre building is sixty feet square and three stories high above the basement; having two wings standing back about eighteen feet from its front, each one hundred feet long by twenty-four feet in depth, and two stories high; terminating in end buildings which project four feet in advance of each wing, and are thirty-one feet four inches in front, by twenty-eight feet four inches in depth, and three stories high, exclusive of the basement. From each of these end buildings, a wing, running south, at right angles with the front, extends twenty-six feet eight inches in length, by twenty-two feet six inches in depth, and corresponding in height with the front wings.

The great extension of front (322 feet), in a building intended for the accommodation of but sixty-five patients, was deemed necessary in order to give to each a separate, well-proportioned room, having all the advantages to be derived from the free admission of light and air. Where the rooms are arranged on both sides of an entry of the usual width, these two essential requisites to health and cheerfulness cannot be commanded; added to which, the patients occupying opposite rooms are very liable to be mutually annoying, and in every respect (unless it be that of saving money), that mode of building for the insane is highly objectionable. On this account, the plan adopted at the Friends' Asylum is worthy of imitation. On one side of the wings are situated the chambers, ten feet square, each having a window, four feet six inches in height by two feet ten inches in width. These rooms open on to the loby, ten feet wide; and directly opposite the door of each room is a window corresponding in size with that in the room. Over each door is fixed a cast-iron sash, thirty-two by twenty inches in size, fitted with a moveable glazed sash, to be opened or shut at pleasure. By this arrangement, a full supply of light and a free circulation of air are secured; and the lobbies being comfortably warmed in cold weather, they afford pleasant places for walking and exercise of different kinds.

Connected with the various buildings described, is a farm of sixty-one acres, the greater part of which is under cultivation, and by giving the patients the opportunity for various agreeable and active out-door employments, affords the most

* In 1834, the Asylum was opened to the public generally.

powerful means for their restoration to health and reason. The woodlands cover about eighteen acres of ground, and are made up principally of the chesnut, beach and oak, affording a deep and delightful solitude and shade. A broad serpentine walk, more than a mile in length, winds throughout them, and a large summer-house and seats in various situations, are provided for the accommodation of the patients. Near the entrance to the woods, and inclosing a small part of them, is a park containing some fine deer.

In the treatment pursued at the Asylum, endeavours are used, so as to combine medical and moral agents, that each shall render the other its most efficient aid, and jointly exert their remedial powers with the greatest certainty and effect. The therapeutical treatment of course varies according to the disease, which, by affecting the brain, disturbs the manifestations of the mind. An accurate account of such treatment and its results, is constantly kept, and at some future day may afford data for ascertaining the relative advantages of the course pursued.

The moral means employed are various. Where it is found necessary, mild and gentle yet firm restraint is imposed, while the earliest gleams of returning reason are watched and cherished.

In the house, there are provided games of different kinds; reading, writing, drawing, &c. The females sew, knit, quilt, &c. The library is furnished with books, periodicals, drawings, &c. Exercise in the open air is always promoted, and the patients encouraged, whenever the weather will permit, to engage in walking and riding. A carriage and horses are always in readiness, morning and evening, for their accommodation. In the lawn fronting the house, is located a circular rail-road about 450 feet in circumference, with a pleasure-car on it, large enough to accommodate two, which is moved by hand. Riding upon this road is a very favorite amusement, and as it is attended with considerable exercise, it is found highly advantageous. Every exertion is made to interest the male patients in gardening, and in the various employments afforded in the cultivation of the farm. The diet of the patient of course varies according to the prescription of the physician, but in general it is plain and nutritious; fresh meat and a variety of vegetables being served up every day. Tea, coffee, and milk, are all abundantly supplied.

| | | | |
|---|---|---|---|
| Whole number of admissions, | | | 634 |
| „ | Men | 331 | |
| „ | Women | 303 | |
| „ | Single | 326 | |
| „ | Married | 234 | |
| „ | Widowers | 17 | |
| „ | Widows | 57 | |
| Of these there were below 20 years of age | | 28 | |
| | From 20 to 30 years | 187 | |
| | „ 30 to 40 „ | 141 | |
| | „ 40 to 50 „ | 126 | |
| | „ 50 to 60 „ | 83 | |
| | „ 60 to 70 „ | 48 | |
| | „ 70 to 80 „ | 15 | |
| | „ 80 to 90 „ | 5 | |
| | „ 90 to 100 „ | 1 | |
| | | | 634 |

Of these 634 admissions, 127 were readmissions granted to 81 individuals, and leaving 507 *persons* who have been under care. The following table shows the duration of the disease at the time of admission of these 507 cases, and the results of treatment:

| Duration. | Number. | Restored. | Much Imp. | Improved. | Stationary. | Remaining. | Died. |
|---|---|---|---|---|---|---|---|
| Less than 1 year | 261 | 152 | 26 | 27 | 18 | 4 | 34 |
| From 1 to 2 years | 57 | 18 | 8 | 8 | 9 | 7 | 7 |
| From 2 to 3 years | 36 | 17 | 3 | 3 | 4 | 5 | 4 |
| From 3 to 5 years | 45 | 14 | 7 | 6 | 9 | 3 | 6 |
| From 5 to 10 years | 47 | 13 | 7 | 3 | 8 | 11 | 5 |
| Over 10 years | 61 | 0 | 7 | 5 | 22 | 13 | 14 |
| Aggregate | 507 | 214 | 52 | 52 | 70 | 43 | 70 |

The proportion of cures in these cases is 42·21 in every hundred; but if we deduct the sixty-one cases which at the time of admission had been deranged over ten years (and which included twenty who either were idiots, or had been imbecile from puberty), five cases complicated with epilepsy, and five which entered the institution with the paralysis peculiar to the insane, it leaves 436 cases, properly subject to treatment, and the cures are in the proportion of 49 in every hundred. The per centage of cures in cases of less than a year's duration, taking the whole twenty-two years, is 58·23. Within the last six years, it has been 66. Nearly all of this class, discharged as "much improved," were almost well; but either pecuniary considerations, or the anxiety of their friends, occasioned their removal as soon as the disease was so far overcome as to render their perfect restoration probable; and in many instances information was afterwards received of their perfect recovery.

Of the seventy deaths, six occurred within a week of the time of their admission; nine within two weeks; seven within three weeks; and three within four weeks; these were mostly cases of acute inflammation of the brain, or its meninges, many of them being brought to the Asylum after all hope of relieving them at home was abandoned. Ten died between a month and a year's residence, and the remainder varying from one year to twenty.

Of the eighty-one patients re-admitted, there were discharged

| | |
|---|---|
| Restored - - - - - | 36 |
| Much Improved - - - - | 4 |
| Improved - - - - - | 6 |
| Stationary - - - - - | 7 |
| Died - - - - | 17 |
| Remaining in the House - - - | 11 |

Twenty-two returned a third time; of whom there were discharged—Restored, 14; improved, 3; stationary, 3; died, 3; and one remains in the house. The other readmissions were of three individuals who, being liable to periodical insanity, have been accustomed to resort to the Asylum at the commencement of an attack, and to remain there until again restored to the use of their reason.

*American Journ. of Med. Science. May,* 1839.

